# Supplementary material for: Exploring Alternative Treatment Choices for Multidrug-Resistant Clinical Strains of Helicobacter pylori in Mongolia
Source: Microorganisms. 2023 Nov 24;11(12):2852. doi: 10.3390/microorganisms11122852 (PMC10745380; doi:10.3390/microorganisms11122852)
Supplement: Supplementary file 1 [file microorganisms-11-02852-s001.zip › microorganisms-2662700-supplementary.pdf]

Supplementary Tables

Supplementary Table S1: Potential genotypes encoded in *gyrA* and *rpoB* genes of CIP-, MOXI-, and RFB-sensitive and -resistant *H. pylori* strains.

Alleles		Sensitive strains		Resistant strains		*p*-value
		n	%	n	%	
gyrA		**38**	**100**	**39**	**100**	
	N87 K/I	1	2.6	15	38	$p = 0.0001$
	A88P	0	0	1	2.5	ns
	D91N/Y/G	1	2.6	12	31	$p = 0.001$
	Total	2	5.2	28	79	ns
rpoB		**227**	**100**	**2**	**100**	
	D530N	0	0	1	50	ns
	R701C	0	0	1	50	ns
	Total	0	0	2	100	ns

In this study, we included sequencing data of 77 quinolone-treated (*gyrA*) and 229 rifabutin-treated (*rpoB*) *H. pylori* strains. Statistical analysis was calculated with Fisher's exact test ($p < 0.05$); ns: no significance.

Supplementary Table S2: Hypothetical mutation alleles from 77 fluoroquinolone-treated (*gyrA* and *gyrB)* and 229 rifabutin-treated (*rpoB*) *H. pylori* strains.

	Hypothetical mutations	Sensitive strains		Resistant strains		*p*-value
		n	%	n	%	
***gyrA* gene**		**38**	**100**	**39**	**100**	
1	V199I	2	5.2	11	28.2	$p = 0.012$
2	N660D	0	0	8	20.5	$p = 0.005$
3	A524V	2	5.2	7	17.9	ns
4	D610N	3	7.8	6	15.3	ns
5	D143E	3	7.8	5	12.8	ns
6	A801V	0	0	4	10.2	ns
7	A379V	1	2.6	4	10.2	ns
8	R486C, A654T, N791K	1	2.6	3	7.6	ns
9	A197V, P595S, H663Y, G712S, Q825L	0	0	2	5.1	ns
10	V13I, A97V, R130K, P151S, A183V, A197T, D210N, R243C, I363T, A375T, M413I, T417I, D440Y, K443R, D464Y, L474F, K478R, S482Y/P, F616L, R625L, K662N, V702I, M776I, P816L	0	0	1	2.5	ns
***gyrB* gene**		**38**	**100**	**39**	**100**	
1	A584V	1	2.6	6		ns
2	I619V	0	0	4	10.2	ns
3	V31I, T51R, D63E	2	5.2	4	10.2	ns
4	A345T, A584T, N685D	1	2.6	3	7.6	ns
5	S240A, A259T, Y670C	0	0	2	5.1	ns
6	E327D	1	2.6	2	5.1	ns
7	E16D, N58S, R127H, T167I, F171L, M194T, E208G, Q213R, E277D, E305D, M572T, K605N, D606E/N, M676T, K726R, S730N, K741R	0	0	1	2.5	ns
***rpoB* gene**		**227**	**100**	**2**	**100**	
1	E470G, L2196P	0	0	1	50	ns
2	S273P	2	0.9	1	50	ns
3	A1173T, A2710P	3	1.3	1	50	ns

ns: no significance.
